# Stochastic geometry analysis of UAV-assisted networks with probabilistic UAV activation

**DOI:** 10.1038/s41598-025-21343-5

**Published:** 2025-10-27

**Authors:** Mahmoud M. Selim

**Affiliations:** 1 Faculty of Engineering, Tanta University, Tanta, 31511 Egypt; 2 Faculty of Engineering, Alamein International University, El-Alamein, 21527 Egypt

**Keywords:** UAVs, Stochastic geometry, Probabilistic activation, Coverage probability, Average achievable rate, Energy efficiency., Engineering, Electrical and electronic engineering

## Abstract

Unmanned aerial vehicles (UAVs) are prominent to modern wireless networks but are severely limited by onboard energy, making continuous operation of a large swarm infeasible. To address this, this paper proposes a probabilistic activation scheme in which UAVs from a larger candidate pool can enter sleep states to conserve energy. Leveraging a tractable probabilistic activation strategy, each UAV is independently switched on with an optimized probability that can be easily derived in closed form. This differentiates our work from earlier heuristic sleep-mode techniques which rely on traffic-threshold rules without analytical guarantees. We thus develop a tractable analytical framework using stochastic geometry to evaluate this scheme, modeling the active UAVs as a 3D Poisson Point Process (PPP) under a probabilistic Line-of-Sight (LoS)/Non Line-of-Sight (NLoS) propagation conditions. Novel analytical expressions are derived for the coverage probability, average achievable rate, and network energy efficiency, with their accuracy rigorously validated by Monte Carlo simulations. Our analysis reveals a fundamental trade-off between network performance and power consumption, demonstrating the existence of an optimal activation probability that maximizes the system performance. This analytical framework enables network operators to optimize activation and transmit power profiles across the entire network. The goal of this optimization is to improve outage probability, ergodic rate, and energy efficiency.

## Introduction

The proliferation of mobile data traffic, driven by the Internet of Things (IoT), massive machine-type communications (mMTC), and enhanced mobile broadband (eMBB), has placed unprecedented demands on terrestrial wireless networks. Traditional fixed base stations (BSs), while forming the backbone of cellular communication, face significant challenges in providing ubiquitous, on-demand, and reliable connectivity, particularly in scenarios involving temporary network augmentation, disaster recovery, or coverage for geographically challenging areas^1^. To address these limitations, unmanned aerial vehicles (UAVs), commonly known as drones, have emerged as a promising technology for future wireless networks (i.e., Beyond 5G (B5G) and 6G)^2^. Specifically, UAV platforms can further facilitate the optimal design of modern wireless sensor networks (WSNs) and wireless-powered communication networks (WPCNs)^3–5^.

UAVs can offer unparalleled deployment flexibility by operating as aerial base stations (ABSs) or mobile relays. Their ability to establish line-of-sight (LoS) communication links by adjusting their altitude, coupled with their inherent mobility and rapid on-demand deployment capabilities, makes them ideal candidates for a plethora of applications. These include providing temporary coverage for large-scale events, restoring communication in post-disaster scenarios where terrestrial infrastructure is compromised, and extending network coverage to rural or remote locations^6^. However, the three-dimensional (3D) mobility and the highly random, dynamic nature of UAV-assisted networks introduce significant analytical challenges. Characterizing network performance, which is governed by the random spatial distribution of UAVs and the complex air-to-ground (A2G) channel, requires a robust mathematical framework capable of capturing these inherent stochastic properties.

Stochastic geometry (SG) has proven to be an indispensable mathematical tool for the modeling, analysis, and design of large-scale wireless networks^7,8^. By modeling the locations of network nodes (e.g., base stations, UAVs and users) as points of a spatial random process, typically a Poisson Point Process (PPP), this framework allows for the derivation of tractable, closed-form or semi-closed-form expressions for key system-level performance metrics such as coverage probability, average achievable rate and network energy efficiency. The power of stochastic geometry lies in its ability to average over all possible network topologies, thereby providing insights that are not specific to a single deterministic deployment but are representative of the network’s overall behavior. The application of stochastic geometry to UAV networks is particularly fitting, as it naturally captures the random placement of UAVs deployed to serve ground users over a wide area^9^.

Foundational studies were among the first to leverage stochastic geometry for the performance analysis of UAV networks. The authors in^10^ derived the coverage probability for a typical user on the ground served by a 2D PPP of UAVs, establishing a fundamental framework and revealing the existence of an optimal UAV altitude that maximizes coverage. This work was extended in^11^ to consider a 3D PPP of UAVs and more generalized fading channels. A finite network of unmanned aerial vehicles serving a given region is considered in^12^, modeling this network as a uniform binomial point process, and the downlink coverage probability of a reference receiver located at an arbitrary position on the ground is thus derived. Investigating the multiple-input multiple-output (MIMO) non-orthogonal multiple access (NOMA) assisted unmanned aerial vehicles (UAVs) networks was conducted in^13^ by utilizing a stochastic geometry model. Analytical expressions for both coverage probability and ergodic rate were derived that confirms that outage probability of NOMA enhanced UAV networks is affected to a large extent by the targeted transmission rates and power allocation factors of NOMA users. Subsequent studies have aimed to enhance model fidelity by integrating more realistic air-to-ground (A2G) channel models, which are critical for precise performance evaluation of UAV networks. For instance, the framework was advanced by Angelou et al. in^14^, who analyzed a network of UAVs modeled as a 3D PPP while adopting a channel that explicitly differentiates between probabilistic LoS and NLoS propagation states.

More recent studies in^15–19^ have employed stochastic geometry to investigate performance of modern UAV-assisted wireless networks. Specifically, authors in^15^ considered uncrewed aerial vehicle (UAV)-assisted maritime low Earth orbit (LEO) satellite communication network to provide coverage for low-end maritime users (MUs), such as buoys in remote ocean regions and derived an approximate yet accurate expression for the success probability. Authors in^16^ investigated a dual-layer heterogeneous communication network assisted by multiple UAVs based on rate-splitting multiple access (RSMA) and derived expressions for coverage probability and area spectral efficiency (ASE). Authors in^17^ considered a CF-mMIMO network containing both ground and aerial users and studied the influence of system parameters on the signal-to-interference-plus-noise ratio (SINR) and rate coverage performance. The study in^18^ estimated both the average and local coverage probability of a wireless network aided by an aerial RIS (ARIS); in particular, the surviving terrestrial base stations (TBSs) are modeled by means of an inhomogeneous Poisson point process, while the UAV is assumed to hover above the disaster epicenter. Finally, authors in^19^ proposed the utilization of a UAV-mounted RIS for data collection and studied the coverage probability in massive Internet of Things networks.

While these studies provide crucial insights into the spatial and channel characteristics of modern UAV-assisted wireless networks, they often assume that all deployed UAVs are continuously active, which presents a major practical challenge. UAVs are energy-constrained platforms always limited by onboard battery capacity. The power consumed for propulsion (i.e., hovering) and communication circuitry often far exceeds the transmit power, making continuous operation of a dense swarm of UAVs energetically infeasible^20^. Addressing this energy constraint has become a pivotal research direction. Some studies have focused on energy-efficient trajectory optimization, which is often computationally complex and may not be suitable for some network adjustments^21^. BS sleeping has been studied for terrestrial networks using stochastic geometry tools and energy-efficiency metrics^22,23^. Other works have proposed UAV sleep/wake-up schedules^24–26^. Specifically, these studies typically optimized height/density or proposed landing/placement strategies without a probabilistic activation rule. However, a gap still exists to find an optimal activation strategy for UAV-assisted networks that strongly depends on different configuration aspects (e.g., UAV altitude, LoS blockage, traffic pattern, UAV density), offering an operator-side decision rule. While these contributions are significant, there remains a gap in the literature for a tractable analytical framework that directly models and evaluates the fundamental performance trade-offs of a simple, scalable, and distributed probabilistic UAV activation scheme using stochastic geometry. Such a scheme, where each UAV in a candidate deployment independently enters an active or sleep state, is a practical approach to conserving energy across the network.

This paper aims to bridge this gap by developing a comprehensive analytical framework based on stochastic geometry to model and analyze a downlink UAV network featuring probabilistic UAV activation. We investigate the impact of this energy-saving strategy on fundamental performance metrics, including coverage probability, average achievable rate, and energy efficiency, under a realistic 3D A2G channel model with probabilistic LoS/NLoS propagation. This work is considered an operator-side insightful analytical framework for UAV-assisted wireless networks. Specifically, stochastic geometry expressions are derived that can be evaluated offline inside a central RAN-intelligent controller for UAV-assisted wireless networks. For a given user-density map, UAV-density map, environmental LoS statistics and energy budget, this controller publishes the optimal on/off probabilistic activation pattern and transmit-power profile to all connected UAVs in the network once every control epoch, so the signaling cost in terms of overhead and latency is negligible in our framework. The salient contributions of this work are as follows:

- We develop a novel and tractable system model for a 3D UAV-assisted wireless network where UAVs are probabilistically activated from a larger candidate PPP deployment. This captures the practical need for energy-efficient operation in large-scale UAV fleets.
- We derive novel, semi-closed-form analytical expressions for the unconditional coverage probability of a typical ground user. The derivation fully incorporates the 3D geometry, the probabilistic LoS/NLoS channel model, and Rayleigh fading for both serving and interfering links.
- Building upon the coverage probability analysis, we derive the analytical expression for the average achievable rate (ergodic spectral efficiency), providing insight into the user’s average data throughput.
- We further formulate a model for the network’s energy efficiency. This allows us to quantify the trade-offs of the activation scheme and identify optimal operating points.
- We validate the accuracy of all our derived analytical expressions through extensive Monte Carlo simulations and present a comprehensive set of numerical results to illustrate the impact of key system parameters-such as UAV density, activation probability, and UAV altitude on network performance.

The remainder of this manuscript is organized as follows. Section System model details the system model. Section Performance analysis presents the detailed analytical derivations for coverage probability, average achievable rate and network energy efficiency. Section Numerical results and discussion provides numerical results and discusses the key performance trade-offs. Finally, Section Conclusion concludes the paper.

## System model

**Fig. 1:**
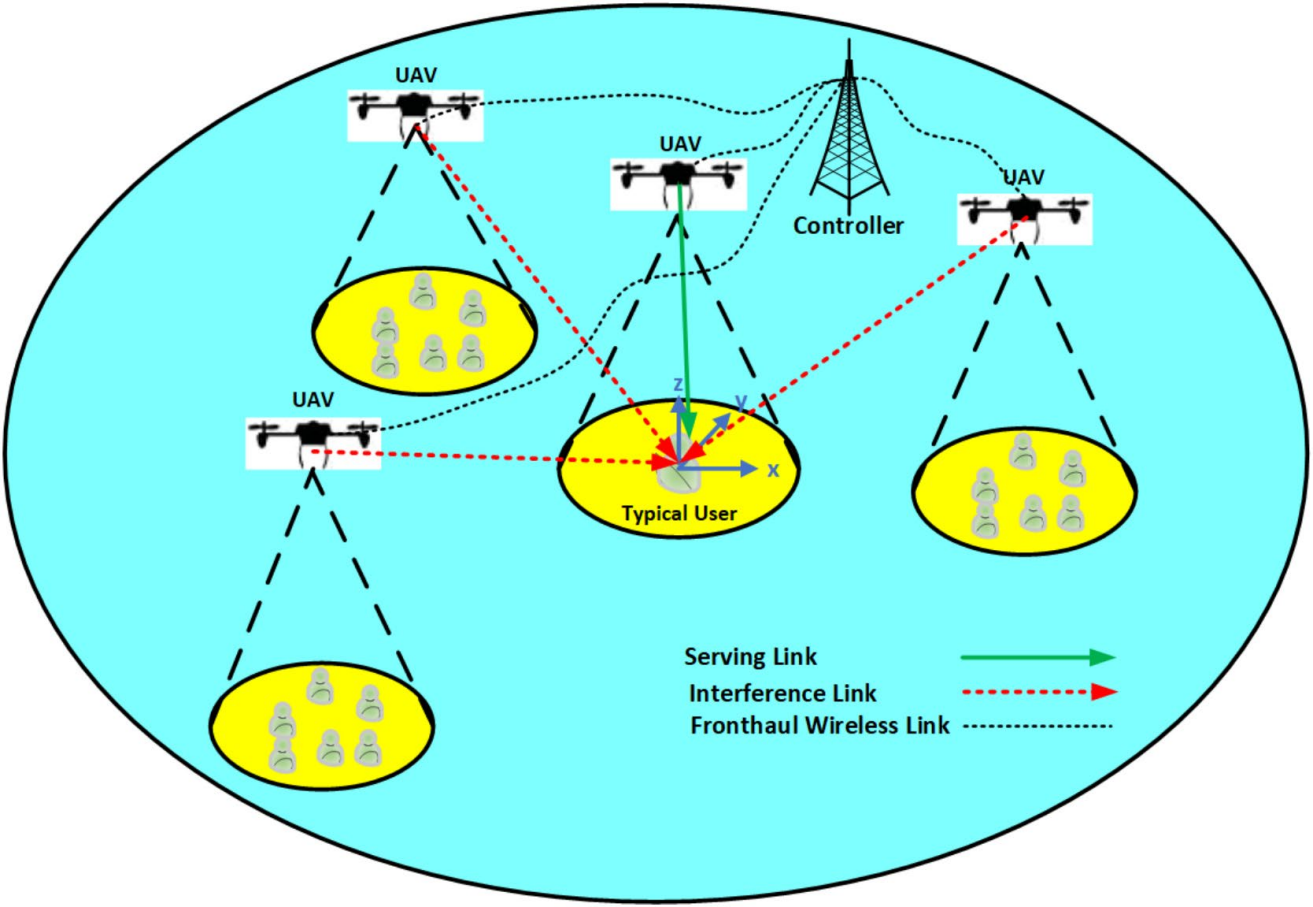
Network architecture.

This section introduces the system architecture and key modeling assumptions adopted for the performance analysis of a UAV-assisted wireless network. We employ a stochastic geometry framework to model the spatial distributions of UAVs and characterize the wireless channel. Performance metrics, such as coverage probability, average achievable rate and network energy efficiency are thus evaluated for a typical ground user.

### Network architecture & activation model

We consider a downlink wireless network supported by UAVs as shown in Fig.1 where UAVs, functioning as aerial base stations (ABSs), provide communication services to users on the ground. The users are clustered around UAVs locations, forming a Matérn Cluster Process (MCP). For analytical tractability, we analyze the performance of a typical stationary user located at its cluster center defined by the reduced Palm distribution of the Homogeneous Poisson Point Process (HPPP) which is fundamental in stochastic-geometry (SG) modelling as it guarantees spatial averages equal time averages under ergodic mobility^27^. Numerous works show that the mean coverage probability and ergodic rate remain unchanged when users move, because mobility doesn’t change the distribution of an infinite PPP; it just shuffles the points around^28,29^. Consequently, only temporal correlations of interference change, which do not affect the optimization of static activation probability in our work^30^. Because the optimization happens every epoch which typically exceeds the user mobility coherence time, snapshots with static typical users provide sufficiently accurate guidance for network operator to optimize network parameters. Extending the framework to include temporal interference correlation and handover costs is further an interesting topic left for future work.

Consistent with standard stochastic geometry analysis and by virtue of Slivnyak’s theorem for Poisson Point Processes (PPPs)^8,27^, this typical user is assumed to be located at the origin $o=(0,0)$ of a two-dimensional (2D) Cartesian coordinate system representing the ground plane. The three-dimensional (3D) locations of the deployed UAVs and their operational status are modeled as follows:

- **Candidate UAV locations**: The ground projections of all deployed UAV locations are spatially distributed according to a Homogeneous Poisson Point Process (HPPP) similar to^31,32^, denoted by $\Phi _{\text {u}}$, with a spatial density $\lambda _{\text {u}}$ [UAVs/$\text {m}^2$]. HPPP yields a stationary, ergodic interferer field and enables closed form analysis using common stochastic geometry analysis tools. Other processes have been used in the literature such as finite Binomial Point Process (BPP) for a known UAVs fleet size, cluster processes (e.g., Poisson Cluster Process (PCP)) for swarms of UAVs, and hard-core processes for minimum distance between deployed UAVs (e.g., Matérn Hard-Core Point Process (MHCPP)). However, we provide here a foundational study to investigate and analyze the performance in terms of coverage and ergodic rate for our proposed probabilistic activated UAV-assisted network, thus we employ HPPP for analytical tractability. Extending our network model for employing other types of processes is left as a future work. All UAVs are assumed to maintain a constant flight altitude of *H* m above their respective ground projections. Thus, a candidate UAV *i* whose ground projection is $X_i \in \Phi _{\text {u}}$ is located at ($X_i,H$) in $\mathbb {R}^3$.
- **Probabilistic activation**: To incorporate energy-saving strategies or load-dependent operation, each candidate UAV in $\Phi _{\text {u}}$ is independently activated with a probability $p_{\text {act}} \in [0,1]$. An active UAV transmits data to its associated user. The set of ground projections of these active UAVs, denoted by $\Phi _{\text {a}}$, also forms an HPPP. This follows from the thinning property of PPPs^33^, and the density of active UAVs is given by $\lambda _\text {a} = \lambda _\text {u}\times p_{\text {act}}$.

The typical user, situated at the origin *o*, establishes a communication link with one of the active UAVs in $\Phi _{\text {a}}$. We adopt a nearest association policy based on 2D ground projection distance: the user connects to the active UAV whose ground projection is closest to it. Let $X_0 \in \Phi _{\text {a}}$ denote the ground projection of this serving UAV. The horizontal distance to the serving UAV is $r_\text {h} = \Vert X_0\Vert$ , where $\Vert .\Vert$ is the Euclidean norm. Given that all active UAVs transmit with the same power $P_\text {u}$ and operate at the same altitude *H*, this association rule is equivalent to selecting the UAV that provides the minimum path loss, and consequently, the strongest average received signal power before considering small-scale fading effect.

### Channel propagation model

The wireless channel between an active UAV and the typical user is characterized by three primary components: distance-dependent path loss, probabilistic Line-of-Sight (LoS)/Non-Line-of-Sight (NLoS) link state, and small-scale fading. The signal power attenuates as a function of the 3D Euclidean distance *R* between the transmitting UAV and the user. If a UAV’s ground projection is at a horizontal distance *x* from the user, the 3D distance is $R = \sqrt{x^2+H^2}$. The distance-dependent path loss is modeled as $L(R,j) = \rho R^{-\alpha _j}$, where $\rho$ is the path loss at a reference distance $d_0=1$m defined as

1 $$\begin{aligned} \rho (d_0) = G_tG_r\left( \frac{\lambda }{4\pi d_0}\right) ^2, \end{aligned}$$

where $G_t, G_r$ and $\lambda$ are the transmit, receive antenna gains and operating wavelength, respectively, and $\alpha _j$ is the path loss exponent corresponding to the link state $j, j\in \{\text {LoS},\text {NLoS}\}$.

The link between a UAV at horizontal distance *x* and the user can be either LoS or NLoS. The probability of a link being LoS, denoted by $P_{\text {LoS}}(x)$, is a function of *x* and *H*. A widely adopted model, e.g.,^10^, is used:

2 $$\begin{aligned} P_{\text {LoS}}(x) = \frac{1}{1 + C_1 \exp \left( -C_2[\theta (x)-C_1]\right) } \end{aligned}$$

where $\theta (x)=\arctan \left( \frac{H}{x}\right) \times \frac{180}{\pi }$ is the elevation angle in degrees and $C_1$ and $C_2$ are positive environmental parameters. The probability of an NLoS link is $P_{\text {NLoS}}(x)=1-P_{\text {LoS}}(x)$. LoS links experience a path loss exponent $\alpha _{\text {LoS}}$, while NLoS links experience $\alpha _{\text {NLoS}}$, where it is generally assumed that $\alpha _{\text {NLoS}} \ge \alpha _{\text {LoS}} \ge 2$. In addition to large-scale path loss, the signal is affected by small-scale fading. We assume all links undergo independent and identically distributed (i.i.d.) Rayleigh fading. Consequently, the channel power gain, *g*, for any link is an exponential random variable with unit mean (i.e., $g \sim \exp (1)$ and $\mathbb {E}[g] = 1$). Log-normal shadowing is omitted to retain closed-form tractability. Prior stochastic geometry analyses show that, when shadowing is sufficiently varied and independent, SINR behavior closely matches that of the underlying Poisson model without it^34^.

### Signal-to-Interference-plus-Noise Ratio (SINR)

All active UAVs in $\Phi _{\text {a}}$ transmit signals with a constant power $P_{\text {u}}$. Furthermore, the receiver at the typical user is subject to Additive White Gaussian Noise (AWGN) characterized by a variance $N_0$ over the communication bandwidth. The quality of the received signal for the typical user, associated with its serving UAV (whose ground projection is $X_0$ and fading gain is $g_0$), is determined by the SINR. Let $R_0 = \sqrt{\Vert X_0\Vert ^2+H^2}$ be the 3D distance to the serving UAV and $\alpha _0$ be its path loss exponent (either $\alpha _{\text {LoS}}$ or $\alpha _{\text {NLoS}}$ depending on the LoS/NLoS state of the serving link). The SINR is given by:

3 $$\begin{aligned} SINR=\frac{P_{\text {u}} g_0 \rho R_0^{-\alpha _0}}{I_{\text {agg}}+N_0} \end{aligned}$$

where $I_{\text {agg}}$ is the aggregate interference power from all other active UAVs. Each active UAV schedules at most one user per resource block; hence intra-cell interference is absent. We further adopted universal frequency reuse (i.e., all simultaneously active UAVs transmit in the same band) because it produces the worst-case aggregate co-channel interference and thus yields conservative design guidelines for network operator. This assumption is often common in stochastic geometry analyses of UAV networks. Under any other spectrum partitioning scheme or multiple-antenna scheme for realizing transmit beamforming, the interference term in our SINR expression in (3) would reduce, so the performance curves reported in Numerical Results Section represent an upper bound on interference and a lower bound on coverage probability and ergodic rate. All our analytical derived expressions in next section remain valid for any carrier frequency $f_c$ and bandwidth slice *B*. A network optimizer (e.g., centralized RAN) can easily post-process our analytical expressions to choose a per-UAV sub-band or allocate orthogonal resources if required for enhancing performance under some system coverage or ergodic rate limits or constraints.

If $X_k \in \Phi _{\text {a}} \setminus \{X_0\}$ is the ground projection of an interfering UAV *k*, $R_k = \sqrt{\Vert X_k\Vert ^2+H^2}$ is its 3D distance to the user, $g_k$ is its fading gain, and $\alpha _k$ is its path loss exponent (LoS or NLoS), then

4 $$\begin{aligned} I_{\text {agg}} = \sum _{X_k \in \Phi _{\text {a}} \setminus {X_0}} P_{\text {u}} g_k \rho R_k^{-\alpha _k}. \end{aligned}$$

## Performance analysis

This section outlines coverage probability, average achievable rate and energy efficiency as key performance metrics used to evaluate the UAV-assisted network and presents the detailed analytical derivation for each metric based on the system model described in Section .

### Coverage probability analysis

Coverage Probability ($\text {P}_{\text {c}}$) is a fundamental metric representing the Quality of Service (QoS). It is defined as the probability that the received Signal-to-Interference-plus-Noise Ratio (SINR) at the typical user exceeds a predefined minimum threshold $\beta$. Mathematically, $P_{\text {c}}=\mathbb {P}(SINR > \beta )$, such that a higher coverage probability indicates a more reliable communication link. The derivation of the unconditional coverage probability $\text {P}_{\text {c}}$ for the typical user at the origin involves averaging the conditional coverage probability over all possible locations of the serving active UAV. Specifically, the coverage probability $\text {P}_{\text {c}}$ can be expressed by conditioning on the serving 3D distance *R* from the user to its serving active UAV, and then averaging over the probability distribution of this distance as follows

5 $$\begin{aligned} P_{\text {c}} = \int _0^\infty \mathbb {P}(\text {SINR} > \beta | R = r) \cdot f_R(r)~dr \end{aligned}$$

such that the 3D distance *R* is related to the horizontal distance random variable $r_{\text {h}}$ by $R = \sqrt{r_{\text {h}}^2+H^2}$ and $f_{r_{\text {h}}}(x)$ is its Probability Density Function (PDF). As established in Section . A, the active UAVs’ ground projections form a Homogeneous Poisson Point Process (HPPP) $\Phi _{\text {a}}$ with density $\lambda _{\text {a}}$. The PDF of the horizontal distance $r_{\text {h}}$ from the origin to the nearest point in $\Phi _{\text {a}}$ is given by^8,27^:

6 $$\begin{aligned} f_{r_{\text {h}}}(x) = 2 \pi \lambda _{\text {a}} x \exp (-\pi \lambda _{\text {a}} x^2), \quad x \ge 0. \end{aligned}$$

We can find the PDF of *R*, denoted as $f_R(r)$, using a change of variables. Since $r_{\text {h}} = \sqrt{r^2-H^2}$, the Jacobian of the transformation is

7 $$\begin{aligned} \frac{dr_{\text {h}}}{dr} = \frac{r}{\sqrt{r^2-H^2}}. \end{aligned}$$

Thus,

8 $$\begin{aligned} f_R(r)&= f_{r_{\text {h}}}\left( \sqrt{r^2-H^2}\right) \left| \frac{dr_{\text {h}}}{dr}\right| \nonumber \\&= 2 \pi \lambda _{\text {a}} \sqrt{r^2-H^2} \exp \left( -\pi \lambda _{\text {a}} \left( r^2-H^2\right) \right) \cdot \frac{r}{\sqrt{r^2-H^2}},\nonumber \\&=2 \pi \lambda _{\text {a}}r\exp \left( -\pi \lambda _{\text {a}} \left( r^2-H^2\right) \right) , r \ge H. \end{aligned}$$

#### Conditional coverage probability

Let $\mathbb {P}(\text {SINR} > \beta | R = r)$ be the probability that the user is in coverage, given its serving active UAV is at 3D distance *r*. Since the serving link experiences Rayleigh fading (i.e., $g_s\sim \exp (1)$) and can be either LoS or NLoS, we can formulate the conditional coverage probability as follows

9 $$\begin{aligned} \mathbb {P}(\text {SINR}> \beta | R = r)&= \mathbb {P}\left( \frac{P_{\text {u}} g_s \rho r^{-\alpha _s}}{I_{\text {agg}}+N_0} > \beta \right) , \end{aligned}$$

10 $$\begin{aligned}&=\mathbb {P}\left( g_s > \frac{\beta (I_{\text {agg}}+N_0)}{P_{\text {u}}\rho r^{-\alpha _s}}\right) . \end{aligned}$$

For a Rayleigh faded desired signal, $\mathbb {P}(g_s > K) = e^{-K}$. Thus, for a given serving link state either LoS or NLoS with path loss exponent $\alpha _s$, we have

11 $$\begin{aligned} \mathbb {P}(\text {SINR} > \beta | R = r) = \mathbb {E}_{I_{\text {agg}},\alpha _s} \left[ \exp \left( -\frac{\beta (I_{\text {agg}} + N_0)}{P_{\text {u}} \rho r^{-\alpha _s}} \right) \right] \end{aligned}$$

Let $s = \frac{\beta r^{\alpha _s}}{\rho P_{\text {u}}}$, thus (11) becomes:

12 $$\begin{aligned} \mathbb {P}(\text {SINR} > \beta | R = r) = \mathbb {E}_{\alpha _s} \left[ \exp \left( -sN_0\right) \cdot \mathbb {E}_{I_{\text {agg}}}\left[ e^{-sI_{\text {agg}}}\right] \right] \end{aligned}$$

where $\mathscr {L}_{I_{\text {agg}}}(s)$ is the Laplace transform of the aggregate interference $I_{\text {agg}}$, defined as $\mathscr {L}_{I_{\text {agg}}}(s) = \mathbb {E}_{I_{\text {agg}}}\left[ \exp \left( -sI_{\text {agg}}\right) \right]$ such that the parameter *s* depends on the serving link’s state either LoS/NLoS and distance *r*. So, the conditional coverage probability is

13 $$\begin{aligned} \mathbb {P}(\text {SINR} > \beta | R = r) = \mathbb {E}_{\alpha _s} \left[ \exp \left( -sN_0\right) \cdot \mathscr {L}_{I_{\text {agg}}}(s)\right] . \end{aligned}$$

The aggregate interference $I_{\text {agg}}$ is the sum of powers from all active UAVs in $\Phi _{\text {a}}$ at horizontal distances ($\Vert X_k\Vert > \Vert X_0\Vert , \forall X_k \in \Phi _{\text {a}} \setminus X_0$) excluding the serving UAV located at horizontal distance $\Vert X_0\Vert$. Due to Slivnyak’s theorem^8^, the interfering field is statistically equivalent to that generated by a PPP $\Phi _{\text {a}}$ over $\mathbb {R}^2$. Each interferer also experiences Rayleigh fading $g_k$ and has a probabilistic LoS/NLoS state determining its path loss exponent $\alpha _k \in \{\alpha _{\text {LoS}}, \alpha _{\text {NLoS}}\}$. Using the probability generating functional (PGFL) of the PPP, the Laplace transform of $I_{\text {agg}}$ is given by^7,8^:

14 $$\begin{aligned} \mathscr {L}_{I_{\text {agg}}}(s) = \exp \left( -\lambda _{\text {a}} \int _{\mathbb {R}^2,\Vert X_k\Vert >\Vert X_0\Vert } (1 - \mathbb {E}_{g_k, \alpha _k}\left[ \exp \left( -s\rho P_{\text {u}} g_k\left( x_k^2+H^2\right) ^{-\alpha _k/2}\right) \right] dx_k \right) \end{aligned}$$

where the expectation is over the fading and LoS/NLoS state of an interferer *k* at ground projection $x_k$. For Rayleigh fading $g_k \sim \exp (1)$, we have $\mathbb {E}_{g_k}\left[ \exp \left( -Ag_k\right) \right] =\frac{1}{1+A}$. Therefore, we have

15 $$\begin{aligned} \mathbb {E}_{g_k, \alpha _k}\left[ \exp \left( -s\rho P_{\text {u}} g_k\left( x_k^2+H^2\right) ^{-\alpha _k/2}\right) \right] = \mathbb {E}_{\alpha _k}\left[ \frac{1}{1+s\rho P_{\text {u}} \left( x_k^2+H^2\right) ^{-\alpha _k/2}}\right] \end{aligned}$$

Averaging over the interferer’s LoS/NLoS state (with $P_{\text {LoS}}(x_k)$ being the LoS probability for an interferer at horizontal distance $x_k$), we can compute expectation in (15) as follows,

16 $$\begin{aligned} \mathbb {E}_{\alpha _k}[.] = P_{\text {LoS}}(x_k) \frac{1}{1 + s\rho P_{\text {u}} \left( x_k^2+H^2\right) ^{-\alpha _{\text {LoS}}/2}} + P_{\text {NLoS}}(x_k) \frac{1}{1 + s\rho P_{\text {u}} \left( x_k^2+H^2\right) ^{-\alpha _{\text {NLoS}}/2}}. \end{aligned}$$

Substituting (16) into (14) and converting to polar coordinates, the Laplace transform in (14) becomes

17 $$\begin{aligned} \mathscr {L}_{I_{\text {agg}}}(s) = \exp \left( -2\pi \lambda _a \int _{\Vert X_0\Vert }^{\infty } \left( 1-\left[ \frac{P_{\text {LoS}}(x_k)}{1+A_{\text {LoS}}} + \frac{P_{\text {NLoS}}}{1+A_{\text {NLoS}}}\right] \right) x_kdx_k\right) , \end{aligned}$$

where $A_{\text {LoS}}= s\rho P_{\text {u}} (x_k^2+H^2)^{-\alpha _{\text {LoS}}/2}$ and $A_{\text {NLoS}} = s\rho P_{\text {u}} (x_k^2+H^2)^{-\alpha _{\text {NLoS}}/2}$. The integral in (17) is typically evaluated numerically.

The final expression for the conditional coverage probability involves averaging over the LoS/NLoS state of the serving link at distance *r* such that

18 $$\begin{aligned} \mathbb {P}(.) = P_{\text {LoS}}(r_{\text {h}})\cdot \exp \left( -\frac{\beta N_0 r^{\alpha _{\text {LoS}}}}{\rho P_{\text {u}}}\right) \cdot \mathscr {L}_{I_{\text {agg}}}\left( \frac{\beta r^{\alpha _{\text {LoS}}}}{\rho P_{\text {u}}}\right) + (1-P_{\text {LoS}}(r_{\text {h}}))\cdot \exp \left( -\frac{\beta N_0 r^{\alpha _{\text {NLoS}}}}{\rho P_{\text {u}}}\right) \cdot \mathscr {L}_{I_{\text {agg}}}\left( \frac{\beta r^{\alpha _{\text {NLoS}}}}{\rho P_{\text {u}}}\right) \end{aligned}$$

#### Unconditional coverage probability

Substituting (8) and (18) (which uses $\mathscr {L}_{I_{\text {agg}}}(s)$ from (17)) into the general formulation (5), we obtain the final expression for $P_{\text {c}}$ (at the top of this page). This expression involves a nested integral structure that requires numerical evaluation.

19 $$\begin{aligned} \begin{aligned} P_c&= \int _H^\infty \bigg [P_{\text {LoS}}\left( \sqrt{r^2-H^2}\right) \times \exp \left( -\frac{\beta N_0 r^{\alpha _{\text {LoS}}}}{\rho P_{\text {u}}}\right) \times \mathscr {L}_{I_{\text {agg}}}\left( \frac{\beta r^{\alpha _{\text {LoS}}}}{\rho P_{\text {u}}}\right) +\\&(1-P_{\text {LoS}}\left( \sqrt{r^2-H^2}\right) )\times \exp \left( -\frac{\beta N_0 r^{\alpha _{\text {NLoS}}}}{\rho P_{\text {u}}}\right) \times \mathscr {L}_{I_{\text {agg}}}\left( \frac{\beta r^{\alpha _{\text {NLoS}}}}{\rho P_{\text {u}}}\right) \bigg ] \times 2 \pi \lambda _{\text {a}}r\exp \left( -\pi \lambda _{\text {a}} \left( r^2-H^2\right) \right) dr. \end{aligned} \end{aligned}$$

### Average achievable rate analysis

Beyond coverage probability, the average achievable rate ($\mathscr {R}$) provides a more comprehensive measure of the typical user’s spectral efficiency, quantifying the average data throughput in (bps/Hz). Thus, the instantaneous achievable rate is given by the Shannon-Hartley theorem^35^. The average achievable rate is then the expectation of this instantaneous rate as follows

20 $$\begin{aligned} \mathscr {R} = \mathbb {E}\left[ \log _2\left( 1+\text {SINR}\right) \right] . \end{aligned}$$

This expectation can be computed using the complementary cumulative distribution function (CCDF) of the SINR, which is precisely the coverage probability $P_{\text {c}}(t)=\mathbb {P}\left( \text {SINR}>t\right)$, where *t* denotes an arbitrary SINR threshold. The relationship is given by:

21 $$\begin{aligned} \mathscr {R} = \frac{1}{\ln (2)}\int _0^{\infty }\frac{P_{\text {c}}(t)}{1+t}dt. \end{aligned}$$

To evaluate $\mathscr {R}$, we substitute the complete analytical expression for $P_{\text {c}}(t)$ (derived in Section .A) into this integral. Recall that $P_{\text {c}}(t)$ itself involves an integral over the serving distance *r*, and the Laplace Transform $\mathscr {L}_{I_{\text {agg}}}(s)$ within $P_{\text {c}}(t)$ also involves an integral over interferer distance $x_k$ for arbitrary interferer *k*. This leads to a multi-level integral for $\mathscr {R}$ as follows,

22 $$\begin{aligned} \mathscr {R}=\frac{1}{\ln (2)}\int _0^{\infty }\frac{1}{1+t}\left( \int _H^{\infty }\mathbb {P}\left( \text {SINR}>t|R=r\right) \cdot f_{R}(r)dr\right) dt. \end{aligned}$$

### Network energy efficiency analysis

A critical performance indicator for battery-powered UAV networks is the energy efficiency (EE), which quantifies the trade-off between achievable data rate and power consumption. We define the network’s energy efficiency as the ratio of the total achievable data rate per unit area to the total power consumed per unit area.

First, we define the Area Spectral Efficiency (ASE), denoted as $\mathscr {A}$,as the aggregate spectral throughput per unit area (bps/Hz/m$^{2}$)^36^. For our downlink with one scheduled user per RB and universal reuse, the ASE can be expressed as the product of the density of active serving nodes, $\lambda _{\text {a}}$, and the average achievable rate per link, $\mathscr {R}$ such that

23 $$\begin{aligned} \mathscr {A} = \lambda _{\text {a}} \cdot \mathscr {R} = (\lambda _{\text {u}} \times p_{\text {act}}) \cdot \mathscr {R} \end{aligned}$$

where $\mathscr {R}$ is given by (22). ASE is further the natural capacity-density key performance indicator (KPI); multiplying ASE by the system bandwidth and geographic area gives the aggregate offered traffic. Moreover, ASE is strongly important for our operator-side optimizer: increasing $p_{act}$ raises $\lambda _a$ (but can reduce $\mathscr {R}$ via stronger interference); the optimiser thus selects $p_{\text {act}}^*$ to balance this trade-off. SG results also explain why, in interference-limited regimes, densification can leave per-user ergodic rate roughly invariant while ASE grows with $\lambda _a$ which is a key rationale for using ASE as an important metric.

The total power consumption is composed of the power expended by all deployed UAVs. The power consumed by a single active UAV, $P_{\text {u}}^{\text {tot}}$ includes three main components; the wireless transmission power $P_{\text {u}}$, a constant operational power $P_{\text {op}}$ required for circuit operations and propulsion (hover) power denoted as $P_{\text {prop}}(V)$. We adopt the following rotary-wing propulsion power model^37^ such that

24 $$\begin{aligned} P_{\text {prob}}(V)=P_0\left( 1+\frac{3V^2}{U^2_{\text {tip}}}\right) +P_{\text {i}}\left( \sqrt{1+\frac{V^4}{4v_0^2}}-\frac{V^2}{2v_0^2}\right) ^{1/2}+\frac{1}{2}d_0\rho sAV^3 \end{aligned}$$

where $P_0$ is blade profile power in hover; $U_{\text {tip}}$ is blade tip speed, $P_{\text {i}}$ is induced power in hover; $v_0$ is mean rotor induced velocity in hover, $d_0$ is fuselage drag coefficient; $\rho$ is air density; *s* is rotor solidity; *A* is rotor disk area and finally *V* is UAV air speed. For steady rotary-wing UAV, we set $V=0$ and therefore, $P_{\text {prop}} =P_0+P_i$. Therefore, the average power consumption per unit area, $\mathscr {P}_{\text {tot}}$, is thus given by

25 $$\begin{aligned} \mathscr {P}_{\text {tot}} = \lambda _{\text {u}} p_{\text {act}} \times \left( P_{\text {u}} + P_{\text {op}}\right) + \lambda _u\times P_{\text {prob}} = \lambda _u(p_{act}(P_u+P_{op})+P_{prop}). \end{aligned}$$

The Energy Efficiency measured in bps/Hz/Watt (or sometimes bit/Hz/Joule) is the ratio of the Area Spectral Efficiency in (23) to the average power consumption per unit area in (25) such that

26 $$\begin{aligned} \text {EE} = \frac{\mathscr {A}}{\mathscr {P}_{\text {tot}}} = \frac{\lambda _a \cdot \mathscr {R}}{\lambda _u(p_{act}(P_u+P_{op})+P_{prop})} \end{aligned}$$

The expression in (26) reveals a fundamental trade-off. The average rate $\mathscr {R}$ is an intricate function of the activation probability $p_{\text {act}}$, as a higher $p_{\text {act}}$ decreases the average serving distance but increases interference. The total power consumption per active UAV, however, is constant. Therefore, the activation probability $p_{\text {act}}$ that maximizes $\mathscr {R}$ will also maximize the network’s energy efficiency. This provides a direct analytical method to determine the optimal energy-efficient operational state for the UAV network.

### Benchmark schemes

To evaluate the effectiveness of our proposed probabilistic activation scheme versus basic alternatives, we have added the following two comparison schemes:

1. ***Fixed activation:*** we compare the optimal activation probability $p^{*}_{\text {act}}$ with the extremes $p_{\text {act}}=1$ (all deployed UAVs are always active) and $p_{\text {act}}=0.5$ (UAVs are sparsely active).
2. ***Distance-threshold activation:*** uavs are only deployed within a bounded radius $R_0$ km for practical aspects. To derive the coverage probability and the ergodic rate for this scheme, we just modify the limits of integration inside the Laplace transform of (17) to be
  27 $$\begin{aligned} \mathscr {L}_{I_{\text {agg}}}(s) = \exp \left( -2\pi \lambda _a \int _{\Vert X_0\Vert }^{R_0} \left( 1-\left[ \frac{P_{\text {LoS}}(x_k)}{1+A_{\text {LoS}}} + \frac{P_{\text {NLoS}}}{1+A_{\text {NLoS}}}\right] \right) x_kdx_k\right) . \end{aligned}$$
  We further modify the limits of integration in (19) to be from *H* to $\sqrt{H^2+R_0^2}$ instead of *H* to $\infty$. We keep everything else with no change.

## Numerical results and discussion

In this section, we present numerical results to validate our derived analytical expressions for coverage probability, average achievable rate and energy efficiency. In addition, we investigate the performance of the proposed UAV-assisted wireless network. We conduct extensive Monte Carlo simulations to obtain the empirical coverage probability, average achievable rate and energy efficiency. These simulation results are then compared against the values obtained by numerically evaluating the analytical formulas derived in Section III. This comparison serves to verify the accuracy of our theoretical framework and allows us to explore the impact of key system parameters-such as the UAV activation probability $p_{\text {act}}$, SINR threshold $\beta$, UAV altitude *H* and UAV density $\lambda _{\text {u}}$ on network performance. Unless otherwise specified, the parameters used for the simulations are listed in Table 1.

**Table 1:** Simulation parameters.

Parameter	Symbol	Value
**Deployment Parameters**		
UAV Candidate Density	$\lambda _{\text {u}}$	10 UAVs/km$^2$
UAV Altitude	*H*	120 m
**Rotary-Wing UAV Parameters**		
Plade Power	$P_0$	14.75 W^37^
Induced Power	$P_i$	41.54 W^37^
**Transmission and Channel Parameters**		
UAV Transmit Power	$P_{\text {u}}$	30 dBm (1 W)
Circuit Operational Power	$P_{\text {op}}$	0.1 W
Transmit & Receive Antenna Gains	$G_t, G_r$	0 dBi^38,39^
Carrier Frequency	$f_c$	2 GHz^38,39^
LoS Path Loss Exponent	$\alpha _{\text {LoS}}$	2.1
NLoS Path Loss Exponent	$\alpha _{\text {NLoS}}$	3.5
LoS Probability Parameter 1	$C_1$	10
LoS Probability Parameter 2	$C_2$	0.1
Small-Scale Fading Model	–	i.i.d. Rayleigh Fading
**Receiver and Performance Parameters**		
Noise Power	$N_0$	$-104$ dBm
SINR Threshold	$\beta$	0 dB
**Monte Carlo Simulation Parameters**		
Number of Iterations	–	10,000
Simulation Area Radius	–	10 km

**Fig. 2:**
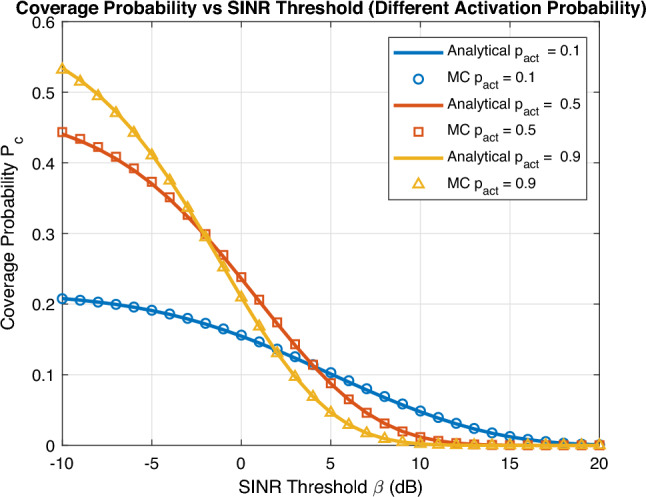
Coverage probability vs SINR threshold.

Fig. 2 validates the analytical expression for coverage probability as a function of SINR threshold ($\beta$) obtained in (19) with Monte Carlo simulations for different UAV activation probability values. It can be easily noted that there is a perfect match between analytical expression and numerical simulations that proves the accuracy of our derived expression. As expected, coverage probability monotonically decrease as SINR threshold ($\beta$) increases. Furthermore, small activation probability $p_{\text {act}}$ is preferable as SINR threshold increases to avoid high interference. As we validated the accuracy of our derived analytical expression in Fig. 2, we will plot only analytical expression in forthcoming figures to to get insights about system performance.

**Fig. 3:**
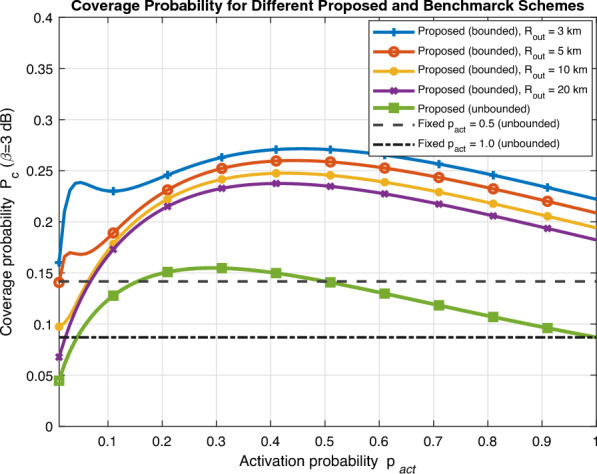
Coverage probability vs activation probability for different schemes.

Fig 3 compares the performance of our proposed unbounded probabilistic activation scheme with two benchmark schemes detailed in the previous section; *fixed activation* and *distance-threshold activation* schemes. In fixed activation scheme, we set $p_{\text {act}}\in \{0.5,1\}$ to represent sparse and full activation, respectively. In distance-threshold scheme, we set $R_0\in \{3,5,10,20\}$ km that represents the bound for interferes radii. We can easily notice that the proposed probabilistic activation scheme has an optimized $p^*_{\text {act}}$ that outperforms the sparse or full activation schemes. Moreover, operator can easily set an outer radius for the proposed scheme to limit the number of interferes and enhance coverage performance based on a design criteria.

**Fig. 4:**
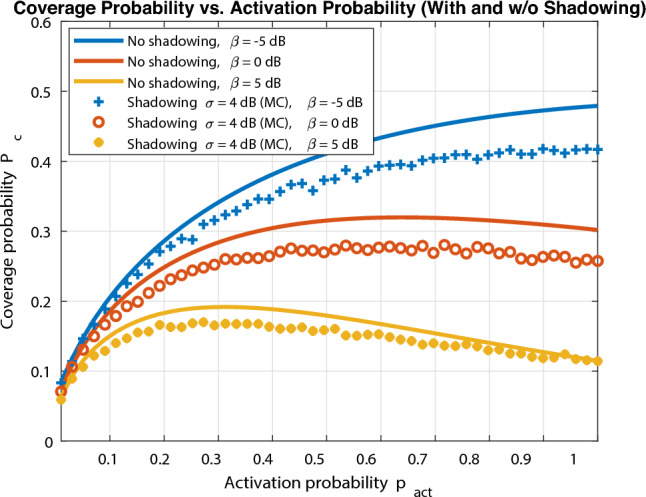
Coverage probability vs UAV activation probability (different SINR threshold).

Fig. 4 shows the coverage probability of a typical user against UAV activation probability $p_{\text {act}}$ for different values of SINR threshold $\beta$. Interestingly, there is always an optimal activation probability $p_{\text {act}}^{*}$ for maximum coverage and this optimal value decreases as SINR threshold increases. This can be explained as mentioned earlier as higher SINR threshold ($\beta$) always limits the number of active UAVs to be turned on to avoid excessive interference. Another interesting insight we can easily notice that excessive activation probability rather results in much smaller coverage probability as the system becomes interference-limited. To further capture the log normal shadowing effect on network performance, we employ Monte Carlo simulations to compute coverage probability for the same proposed probabilistic activation wireless network with log-normal shadowing with standard deviation $\sigma =4$ dB. It can be easily noticed that shadowing does not affect the optimal design of activation probability, yet it results in a decrease in coverage probability. It is noted that coverage degradation becomes smaller as SINR threshold increases and this effect can be compensated by setting a margin by the operator without any further change of optimal activation design.

**Fig. 5:**
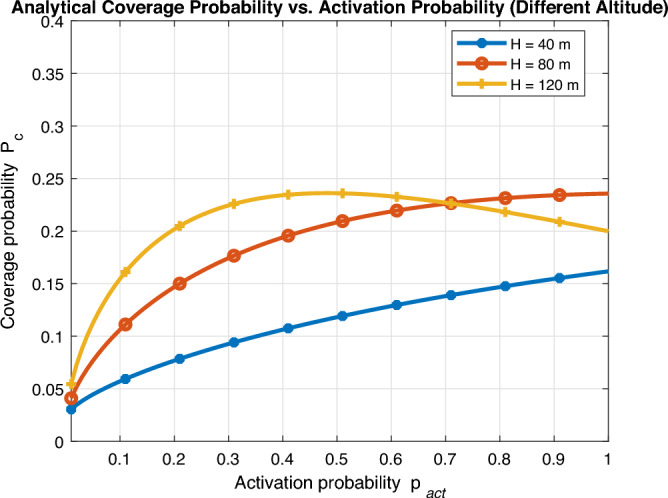
Coverage probability vs UAV activation probability (different UAV altitude).

We further show the performance of coverage probability against UAV activation probability for distinct values of UAV altitude *H* in Fig. 5. It is evident that the optimal activation probability also decreases as UAV altitude increases. This can be explained as higher UAV altitude automatically results in a higher probability of LoS and better channel condition. This automatically leads to better coverage even with a smaller number of UAVs turned on.

**Fig. 6:**
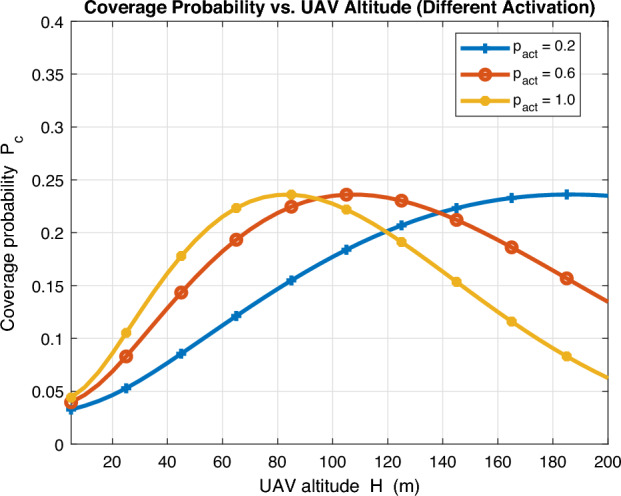
Coverage probability vs UAV altitude.

The impact of varying UAV altitude *H* on coverage probability is investigated in Fig. 6 for different levels of activation probability. One key insight can be observed as high increase in UAV altitude *H* can drastically decrease coverage probability for high level of activation probability as the typical user is then susceptible to excessive number of interfering LoS UAVs. Additionally, the optimal UAV altitude $H^*$ gradually decreases as the activation probability level increases to avoid excessive interference from LoS interfering UAVs.

**Fig. 7:**
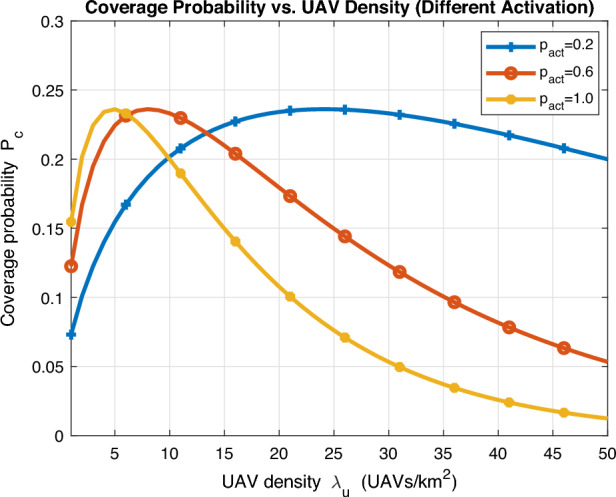
Coverage probability vs UAV density.

We further investigate the impact of UAV density $\lambda _u$ on coverage probability for different activation probability in Fig. 7. Noticeably, an optimal UAV density $\lambda _u^*$ exists which gradually decreases as activation probability increases. The reason is that operator does not need excessive number of deployed UAVs when activation probability is high and vice versa. A key insight can be obtained as further increase in UAV density can drastically deteriorate coverage performance for high activation probability as interference becomes intolerable at the typical user. To further clarify, in classical stochastic geometry analysis with universal frequency reuse, SIR coverage is invariant to density as interference dominates. This is not the case with LoS/NLoS propagation as in UAV-assisted networks. Density invariance disappears and densifying increases LoS probability and interference in a non-monotone way, so an optimal active density $p_{\text {act}}^*$ emerges that our optimizer computes based on altitude, environment and network configuration parameters. From operator-side, when $\lambda _{\text {u}}$ increases, the optimizer can reduce $p_{\text {act}}$ to keep $\lambda _{\text {a}}$ near its optimum or raise $p_{\text {act}}$ to exploit higher ASE, depending on the coverage objective.

**Fig. 8:**
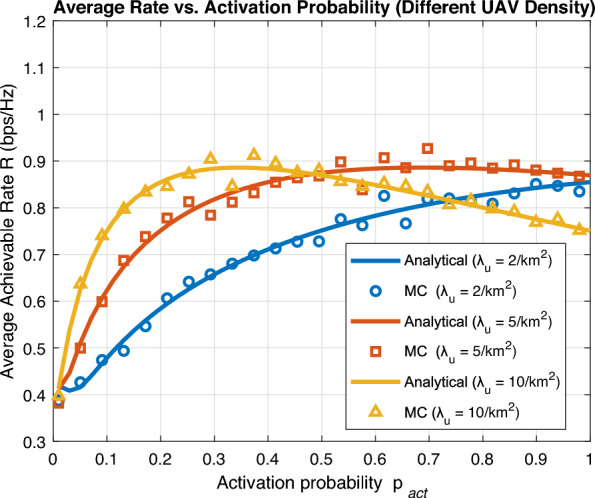
Average achievable rate vs UAV activation probability.

In Fig. 8, we validate the derived analytical expression for average achievable rate in (22) with Monte Carlo simulations. Noticeably, an almost perfect match exists and the accuracy of the derived analytical expression is thus proved. We can further notice that the optimal activation probability $p_{\text {act}}^*$ decreases as the number of deployed UAV per unit area increases. Interestingly, it is required to activate all deployed UAVs for small UAV density to get optimal performances. This is typically explained as there is always a tradeoff between activating a large number of deployed UAVs that may encounter excessive interference and activating a small number of deployed UAVs that may encounter insufficient coverage.

**Fig. 9:**
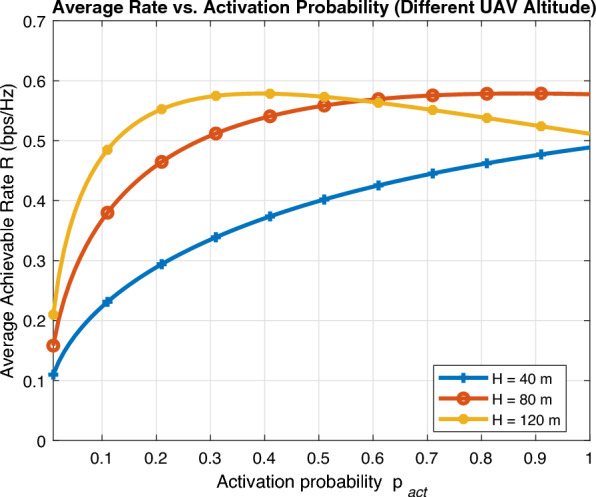
Average achievable rate vs UAV activation probability (different UAV altitude).

We further employ analytical expression of average achievable rate to obtain key insights of system performance. In Fig. 9, we investigate average achievable rate against activation probability for different UAV altitude *H*. A key insight observed is that optimal activation $p_{\text {act}}^*$ gradually decreases as UAV altitude increases. Lower UAV altitude *H* necessitates activating larger number of deployed UAVs and vice versa to preserve system performance.

**Fig. 10:**
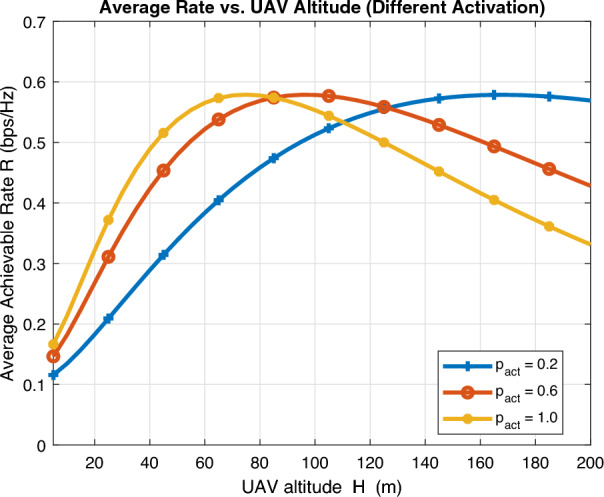
Average achievable rate vs UAV altitude.

**Fig. 11:**
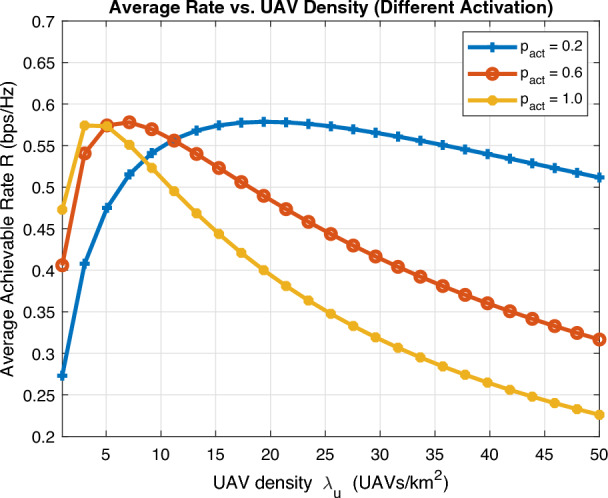
Average achievable rate vs UAV density.

Figures 10 and 11 investigate the performance of average achievable rate against UAV altitude *H* and UAV density $\lambda _u$, respectively, for different activation probability. A key insight can be observed as lower activation probability requires extra UAV altitude for optimal rate to realize LoS channel while this rate drastically decreases as UAV altitude increases for large activation probability due to intolerable interference. Furthermore, increasing UAV density results in dramatic decrease in average achievable rate for high activation probability also due to intolerable interference and there is always an optimal operating point that compromises this tradeoff.

**Fig. 12:**
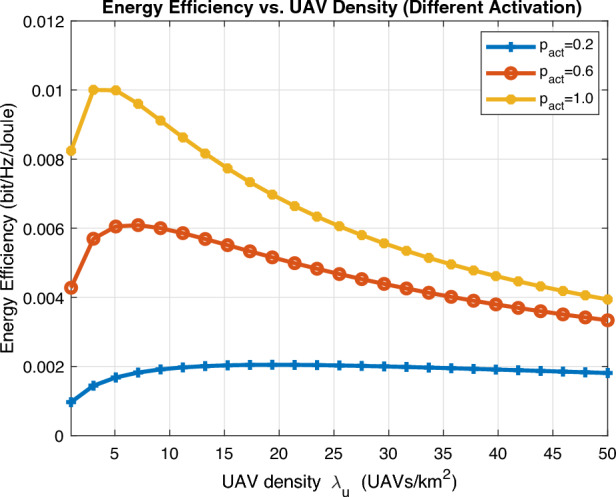
Energy efficiency vs activation probability.

We finally show in Fig. 12 the energy efficiency (EE) analytical expression in bit/Hz/Joule derived in (26) against UAV density $\lambda _\text {u}$ for different activation probability $p_{\text {act}}$. It is worth mentioning that the proposed scenario is interference-limited so increasing transmit power $P_{\text {u}}$ is insignificant. As expected, high energy efficiency is affordable by high activation only for small UAV density, yet energy efficiency dramatically decreases as UAV density highly increases. Moreover, moderate activation probability is further preferred as UAV density increases.

## Conclusion

In this paper, we developed a comprehensive analytical framework using stochastic geometry to investigate the performance of a UAV-assisted wireless network employing a novel probabilistic activation scheme for preserving energy. We derived tractable analytical expressions for the coverage probability, average achievable rate, and network energy efficiency under a realistic channel model. The accuracy of our theoretical framework was rigorously verified against extensive Monte Carlo simulations. Our results unveil the fundamental trade-off between network performance and power consumption, demonstrating that while coverage and ergodic rate initially improve with a higher activation probability or UAV density, they drastically deteriorate after that due to escalating co-channel interference. Crucially, we prove the existence of optimal values for activation probability, UAV altitude and UAV density that maximizes the network’s performance metrics, confirming that an “always-on” deployment is a highly suboptimal strategy. This framework provides critical design insights for dimensioning sustainable and effective UAV communication systems. Extending our framework to encounter load-balancing association and incorporating intra-cell multi-user interference (e.g., power-domain NOMA or MU-MIMO transmission) necessitates a new SINR analysis, which we leave for future work.

## Data Availability

No datasets were generated or analysed during the current study.
